# Characterization of cultivated murine lacrimal gland epithelial cells

**Published:** 2012-05-12

**Authors:** Shinya Kobayashi, Tetsuya Kawakita, Motoko Kawashima, Naoko Okada, Kenji Mishima, Ichiro Saito, Masataka Ito, Shigeto Shimmura, Kazuo Tsubota

**Affiliations:** 1 Department of Ophthalmology, Keio University School of Medicine, Tokyo, Japan; 2 Department of Pathology, Tsurumi University, Tokyo, Japan; 3 Department of Regeneration and Development, National Defense Medical College, Tokyo, Japan

## Abstract

**Purpose:**

To date, mouse lacrimal gland epithelial cells have been cultured successfully but only in cases involving newborn mouse lacrimal glands. In this work, we attempted to cultivate and characterize adult mouse lacrimal gland epithelial cells.

**Methods:**

Lacrimal glands were removed from newborn mice (C57B/6) and isolated lacrimal gland epithelial cells were seeded onto tissue culture treated or low adherent culture dishes in Cnt-07 culture medium with or without cholera toxin. Cultivated cells were characterized by immunostaining with pan-cytokeratin, α-smooth muscle actin, and lactoferrin antibodies. Lacrimal gland cells from 7-week-old green fluorescent protein (GFP) and non-GFP (C57B/6) mice were mixed and seeded onto uncoated dishes to assess sphere-forming efficiency. Cells were also seeded onto 3T3 cell feeder layers to assess colony forming efficiency.

**Results:**

Lacrimal gland epithelial cells were selectively cultured with cholera toxin, and cell type was verified by pan-cytokeratin and α-smooth muscle actin immunostaining. Sphere formation from single cells of adult mice was observed using specific medium and low adherent culture dishes. These cells could also undergo colony formation on 3T3 feeder cells.

**Conclusions:**

Adult mouse lacrimal gland epithelial cells were successfully cultivated in cholera toxin-containing medium, and were observed to form spheres from single cells.

## Introduction

Dry eye is a multifactorial disease often caused by a decrease in secretory function in the lacrimal gland. Dry eye diseases are treated by application of artificial tears, but this treatment only provides transient relief. In severe dry eye, lacrimal gland dysfunction may lead to keratinization of the ocular surface, which may cause severe visual disturbance. Once the lacrimal gland is atrophied or injured, the condition may be irreversible, and recovery of function is rare. In a few cases, lacrimal gland tissues regenerate and their functions are restored.

Stem cells in adult tissues have been extensively studied because of their wide-ranging potential clinical use. Several studies on salivary and mammary glands have shown that stem/progenitor cells exist in these tissues, and are involved in their regeneration [1,2]. However, there are few reports regarding stem cells in the lacrimal gland [3-5]. Several models of cultured lacrimal gland cells have been established to better understand their physiology and pathophysiology [6-16]. Primary cultures of rabbit lacrimal glands could proliferate on plastic, but exhibited morphological differentiation only weakly resembling what was found in vivo [17,18]. Rat lacrimal gland epithelial cell suspension cultures displayed a differentiated acini-like morphology, which was only maintained by the presence of a specific secretagogue [19]. However, these culture systems were only partially defined because of the inclusion of serum in the culture medium. The use of serum-rich media impedes studies of the effects of growth factors, cytokines, and hormones on morphogenesis, growth, and functional differentiation. Ueda et al. [17] reported that primary cultures of mouse lacrimal glands could proliferate in medium without serum. However, newborn mice were used for these animal lacrimal gland culture studies. Because the lacrimal gland of the newborn is very small in comparison with the adult gland, many lacrimal glands from newborns are required for culture experiments.

Establishment of long-term cultures of newborn and adult mouse lacrimal glands would be important for future research on ocular disorders such as dry eye. In this study we attempted to establish long-term cultures of newborn and adult mouse lacrimal gland epithelial cells.

## Methods

### Tissue preparation and cell cultures

C57B/6 mice (CLEA Japan, Tokyo, Japan) aged 1–3 days (newborn), male 7-week-old (adult), and male C57B/6-Tg(CAG-EGFP) mice (green fluorescent protein (GFP); Nihon SLC, Hamamatsu, Japan) were used in accordance with the guidelines in the ARVO Statement for the Use of Animals in Ophthalmic and Vision Research. The mice were euthanized with sodium pentobarbital (Somnopentyl; Kyoritsu Seiyaku Co. Ltd., Tokyo, Japan) and the exorbital lacrimal glands were dissected. After connective tissue was removed, the glands were dissociated by mincing and collagenase digestion as described previously [20], with the following modifications. Briefly, the glands were decapsulated using a fine forceps in Dulbecco’s Modified Eagle’s Medium (DMEM; Invitrogen, Carlsbad, CA) with 10 mM HEPES (Invitrogen) and 10% fetal calf serum (FCS). After mincing, the tissues were digested with DMEM containing 750 U/ml collagenase type I (Wako, Osaka, Japan), 500 U/ml hyaluronidase type I-S (Sigma-Aldrich, St. Louis, MO), 0.01% DNase I (Roche Diagnostics, Indianapolis, IN), and 10% FCS at 37 °C for 60 min with vigorous shaking. Digested cells were filtered through a 100 μm mesh nylon cell strainer (BD Biosciences, Franklin Lakes, NJ). Cells that were passed through the strainer were centrifuged at 460× g for 20 s to remove the supernatant. Cells were resuspended in DMEM with 10 mM HEPES and 10% FCS, and centrifuged at 460× g for 20 s. After removing the supernatant, the cells were resuspended in cold phosphate-buffered saline (PBS) and centrifuged at 460× g for 20 s. The cells were digested with 0.05% trypsin-0.02% EDTA (Invitrogen) and 0.01% DNase I at 37 °C for 20 min. Digested cells were plated onto type I collagen coated culture dishes or plates (Asahi Techno Glass, Tokyo, Japan) and cultured in epidermal keratinocyte medium (CnT-07; CELLnTEC Advanced Cell Systems, Bern, Switzerland) supplemented with growth supplements as provided by the manufacturer, plus 10 ng/ml human recombinant epidermal growth factor (EGF; Invitrogen), 0.25% penicillin–streptomycin (PS; Invitrogen), and with or without 100 ng/mL cholera toxin (CT; List Biologic Laboratories Inc., Campbell, CA; abbreviated; CnT-07 with or without CT), and 10% FCS at a density of 2.0×10^4^ cells/cm^2^, at 37 °C, in 5% CO_2_. After 2 days, the medium was changed to FCS free CnT-07 with or without CT medium, which was replaced every 2–3 days. When subconfluent after approximately 10 days, the epithelial cells were subcultured (TrypLE Express; Invitrogen) at a density of 1.0×10^4^ cells/cm^2^. The procedure was repeated until passage (P) nine.

### Histology and immunohistochemistry

Some isolated newborn and adult mouse exorbital lacrimal glands were embedded in optimal cutting temperature (OCT; Ted Pella, Inc. and PELCO International, Redding, CA) compound and frozen in liquid nitrogen. Frozen sections were stained with hematoxylin-eosin (H&E) for histologic examination. The lacrimal gland cells were cultured in 24 well plates (1.5×10^4^ cells/well) and fixed with ice-cold methanol for immunostaining with pan-cytokeratin (pan-CK), α-smooth muscle actin (α-SMA), lactoferrin, cytokeratin 8, and cytokeratin 14 (CK8 and CK14) antibodies. After background staining was blocked with 10% normal donkey serum, the cells were treated with the following monoclonal primary antibodies: anti–pan-CK antibody (Abcam, Cambridge, UK), anti–α-SMA antibody (Abcam), anti-lactoferrin antibody (Sigma-Aldrich), anti-CK8 (Progen Biotechnik, Heidelberg, Germany), and anti CK14 (Santa Cruz Biotechnology, Santa Cruz, CA). The cells were then treated with cyanine 3 (Cy3) or fluorescein isothiocyanate (FITC)-conjugated secondary antibodies (Jackson ImmunoResearch, West Grove, PA). Cell nuclei were counterstained with 4',6’-diamino-2-phenylindole (DAPI, 1 μg/ml; Dojindo Laboratories, Tokyo, Japan).

### Three-dimensional culture, sphere culture, and colony-forming assay

Mouse lacrimal gland epithelial cells were grown to a subconfluent state in monolayer cultures, and were dissociated by TrypLE Express treatment. For three-dimensional cultures, the dissociated cells were embedded in Cellmatrix Type I-A (Nitta Gelatin, Osaka, Japan) according to the manufacturer’s instructions at a density of 2.6×10^4^ cells/cm^2^, and maintained in FCS-free CnT-07 with CT medium for 7 days.

Sphere culture was performed as described previously [21], with some modifications. Dissociated epithelial cells were mixed with 7.5×10^3^ cells and suspended in 1:1 Matrigel (BD Biosciences) and FCS-free CnT-07 with CT medium in a total volume of 80 µl. Each sample was plated around the rim of a well in a 12 well culture plate and allowed to solidify for 20 min before 2 ml of FCS-free CnT-07 with CT medium was added. The medium was changed every 3 days. After 10 days, spheres were collected by Cell Recovery Solution (BD Biosciences) according to the manufacturer’s protocol, and monolayer and three-dimensional cultures were attempted. For passage of spheres, Matrigel was digested by incubation in dispase (Invitrogen) at 37 °C for 30 min. Digested cultures were pelleted and incubated in 1 ml of FCS-free CnT-07 with 10% collagenase at 37 °C for 30 min. Samples were again pelleted and incubated in TrypLE Express media at room temperature for 10 min, passed several times through a 26 gauge syringe, and passed over a 100 μm mesh nylon cell strainer.

NIH-3T3 cells were suspended in DMEM with 10% FCS and 1% PS, seeded onto a 6 well plate (3.0×10^5^ cells/well), and treated with mitomycin C (MMC; Nacalai Tesque, Kyoto, Japan) for 2 h. A total of 1×10^3^ dissociated lacrimal gland epithelial cells were seeded onto the top of the MMC-treated NIH-3T3 feeder layer and maintained in 1:1 FCS-free CnT-07 with CT medium:DMEM-F12 with 10% FCS, and 1% PS. After 12 days, the cells were fixed with 4% paraformaldehyde and stained with 1% rhodamine B (Wako) in distilled water.

## Results

### Effect of CT on newborn mouse lacrimal gland epithelial cell culture

**Figure 1:**
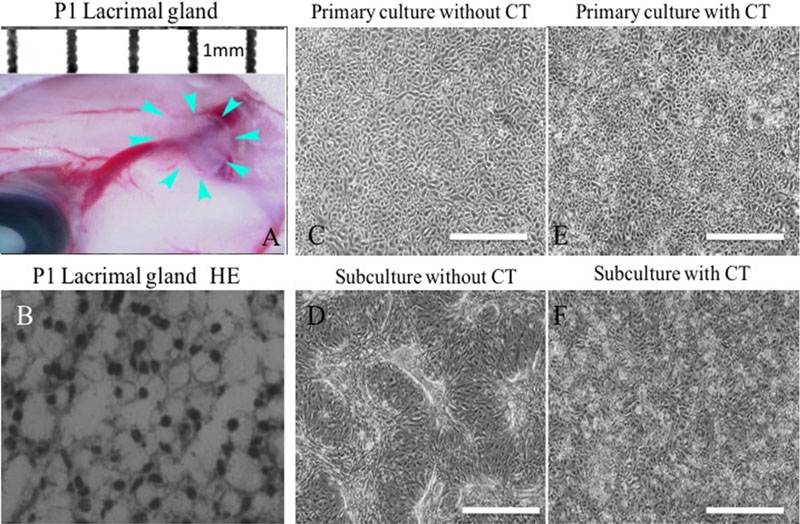
Effect of cholera toxin (CT) on newborn mouse lacrimal gland epithelial cell cultures. Lacrimal gland (**A**) and H&E staining (**B**) of the lacrimal gland. *Arrows*: sections used for culturing. Primary cultures of lacrimal gland epithelial cells after 10 days with (**E**) or without (**C**) CT. Cells at P1 after 2 days with (**F**) or without (**D**) CT. Scale bars, 100 µm.

**Figure 2:**
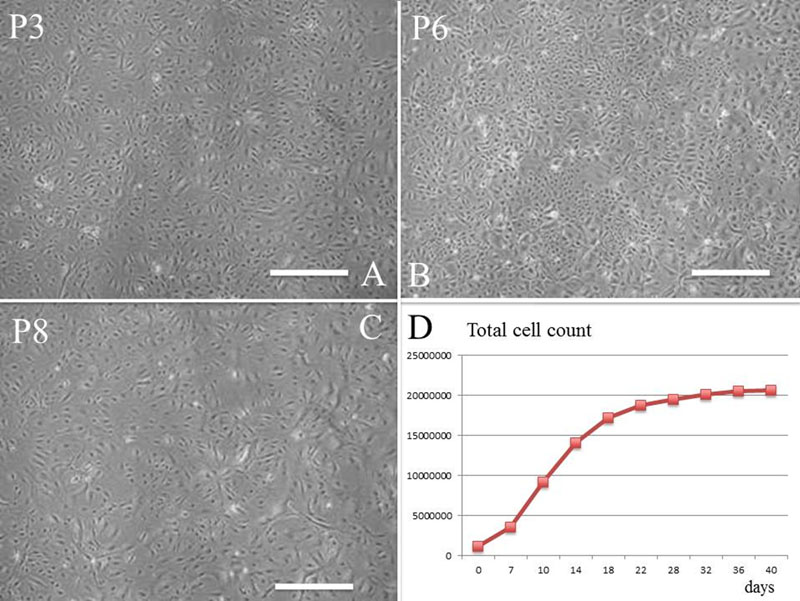
Subcultures of newborn mouse lacrimal gland epithelial cells with CT. Cells at P3 (**A**), P6 (**B**), and P8 (**C**) after 2 days. **D**: The total number of expanded lacrimal gland epithelial cells is shown. Scale bars, 100 µm.

A newborn mouse lacrimal gland is shown in Figure 1A. H&E staining of isolated tissue (Figure 1A, arrow) revealed acinar structures and ducts (Figure 1B). Lacrimal gland cells isolated by trypsin treatment were cultured with or without CT (Figure 1C–F). Without CT, the cells formed a confluent monolayer of polygonal cells in primary cultures (Figure 1C), and morphology was remarkably altered after subculture (Figure 1D). Cell morphology after subculture was maintained in the medium with CT (Figure 1E, F). Furthermore, correct morphology was maintained even at P3 (Figure 2A), P6 (Figure 2B), and P8 (Figure 2C), and subculture was possible until P9 (Figure 2D) while still maintaining cellular morphology.

**Figure 3:**
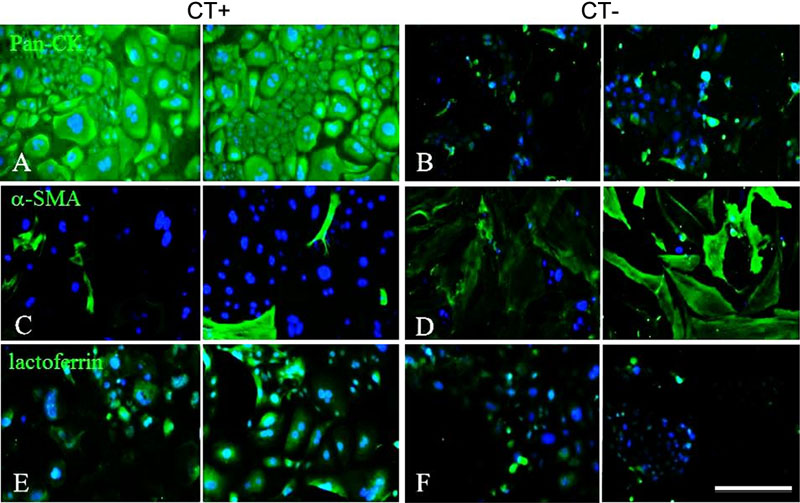
Immunohistochemistry of newborn mouse lacrimal gland epithelial cells at P3 after 2 days. Immunohistochemistry for pan-CK (**A**; fluorescein), α-SMA (**C**; fluorescein), and lactoferrin (**E**; fluorescein) with CT, and for pan-CK (**B**), α-SMA (**D**), and lactoferrin (**F**) without CT. Scale bars, 100 µm.

Figure 3 shows the results of immunostaining of lacrimal gland cell cultures with or without CT. With CT, the cells were pan-CK positive (Figure 3A) and α-SMA negative (Figure 3C), confirming the existence of epithelial cells. In addition, some cells were lactoferrin positive (Figure 3E). In the medium without CT most of the cells were pan-CK negative (Figure 3B) and α-SMA positive (Figure 3D), while a few were lactoferrin positive (Figure 3F). These results suggest that medium with CT could maintain lacrimal gland cellular morphology and epithelial phenotype. For further cell culture experiments, medium with CT was used.

### Three-dimensional and sphere cultures of newborn mouse lacrimal gland epithelial cells

**Figure 4:**
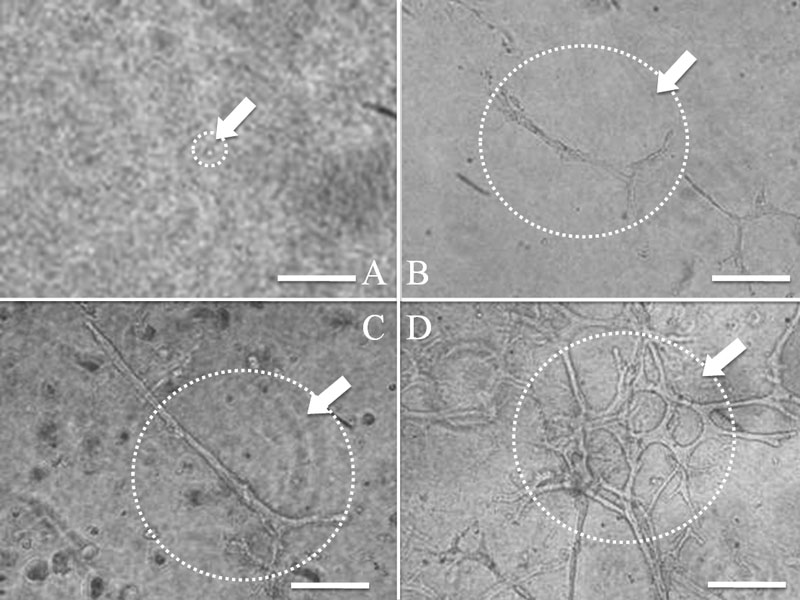
Time-dependent change of three-dimensional culture at a density of 2.6×10^4^ cells/cm^2^. Microscopic images at 0 (**A**), 2 (**B**), 4 (**C**), and 6 days (**D**) are shown. From a single cell (**A**), elongation occurred to generate structures (**B** and **C**), and finally complex structures were observed (**D**). Scale bars, 100 µm.

**Figure 5:**
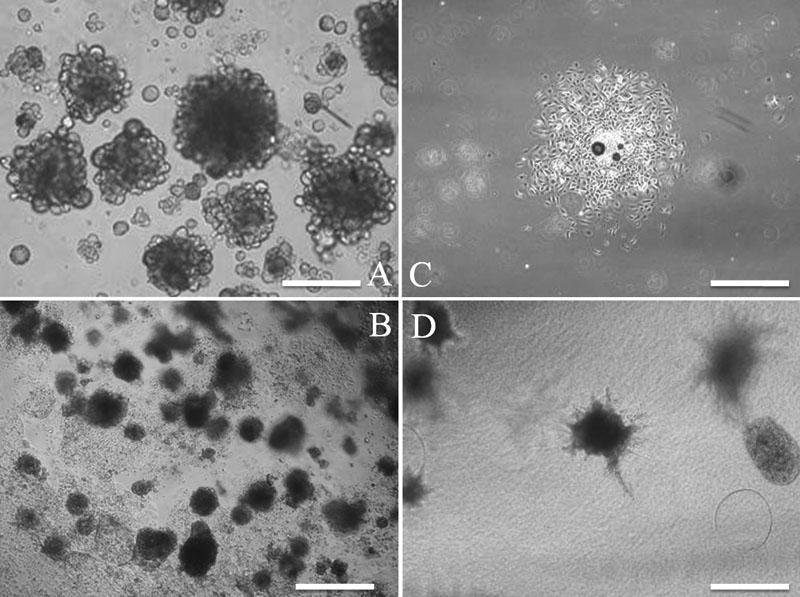
Sphere culture of newborn mouse lacrimal gland epithelial cells. Spheres were clearly formed after 10 days (**A**). When spheres was dissociated with trypsin/EDTA and subcultured for 10 days, sphere formation was regenerated (**B**). When spheres were placed in tissue culture treated dishes for 4 days, the spheres attached to the dishes and cells were expanded (**C**). When isolated spheres were cultured and embedded in collagen type I for 8 days, bundle-like cells expanded from the spheres (**D**). Scale bars, (**A**) 100 µm; (**B**) 200 µm; (**C**, **D**) 400 µm.

The branching structure was most prevalent at a density of 2.6×10^4^ cells/cm^2^, when epithelial cells were embedded in Cellmatrix Type I-A and cultured at various cell densities (preliminary study; data not shown). Figure 4 shows time-dependent changes of three-dimensional cultures. After 2 days, formation of branching structures was evident (Figure 4B), and the branching structure became more complex in a time-dependent manner (Figure 4C,D). Moreover, spheres were formed by embedding in Matrigel (Figure 5A), and subculturing was possible (Figure 5B). When the spheres were isolated for plate cultures, those that had an epithelial-like appearance from the surrounding sphere proliferated after 4 days (Figure 5C). When the isolated spheres were embedded in Cellmatrix, the branching structure was formed 8 days after culture (Figure 5D). All of the cells in the spheres were CK14 positive and α-SMA negative (data not shown).

### Adult mouse lacrimal gland epithelial cell culture

**Figure 6:**
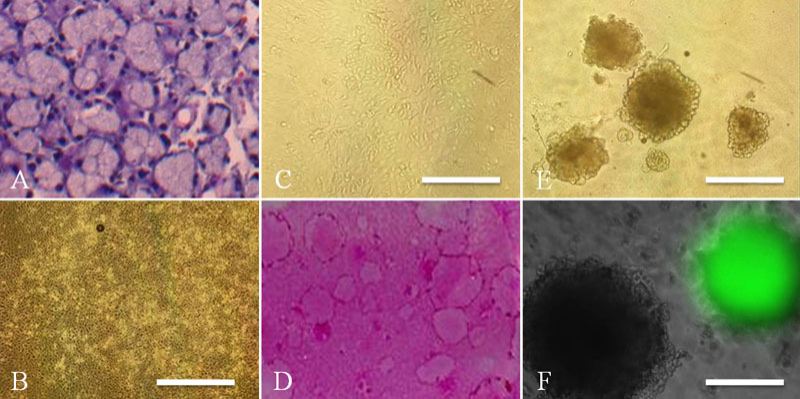
Adult mouse lacrimal gland epithelial cell cultures with CT. H&E staining of the adult lacrimal gland showing regular acinar unit structures (**A**). Primary cultures of adult lacrimal gland epithelial cells after 10 days showing a cobblestone structure (**B**). After subculture, cells at passage 1 showed similar cell morphology (**C**). Clear colony formation was generated on 3T3 feeder layers after 12 days (**D**). Spheres were also generated from adult lacrimal gland cells after 10 days (**E**), and were generated from GFP positive or GFP negative cells (**F**). Scale bars, (**B**, **C**, **E**) 100 µm; (**F**) 50 µm.

**Figure 7:**
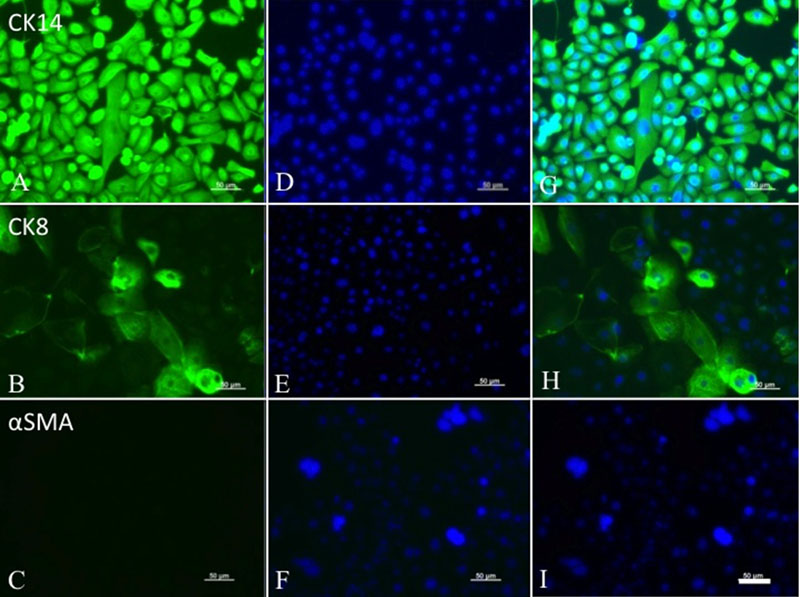
Adult mouse lacrimal gland spheres were treated by trypsin/EDTA, and cultured for further epithelial characterization. All sphere-forming cells showed expression of epithelial cell markers, CK14 (**A**), confirmed by nuclear staining (**D**), and merged images (**G**). CK8 was partially positive in these cells (**B**), confirmed by nuclear staining (**E**), and merged images (**H**). However, no positive expression of α-SMA was observed (**C**, **F**, and **I**). Scale bars, 50 µm.

Figure 6A shows H&E staining of the adult mouse lacrimal gland. Acinar structures and ducts were clearly observed. Similar to newborn mice, the lacrimal gland cells of adult mice formed a confluent monolayer of polygonal cells in primary cultures (Figure 6B), and morphology was maintained after subculture (Figure 6C). Furthermore, these cells had colony- and sphere-forming efficiency (Figure 6D, E). As shown in Figure 6F, homogeneous GFP positive or GFP negative spheres were found 10 days after culture, and >90% of spheres were homogeneous. All of the cells in the spheres were CK14 positive and α-SMA negative, and also included CK8 positive cell populations (Figure 7).

## Discussion

In this study, we report successful culturing of newborn and adult mouse lacrimal gland epithelial cells by adding CT to the culture medium. The primary lacrimal gland epithelial cells could form spheres from single cells, which could then be subcultured on plastic dishes.

We hypothesized that lacrimal glands have their own tissue-specific stem cells that generate three differentiated phenotypes, acinar epithelial cells, myofibroblasts, and ductal epithelial cells. Previous studies using adult mouse corneal epithelial cells found that adding CT to the culture medium resulted in cells that could be cultured long-term without fibroblast contamination [22]. In contrast, lacrimal glands contain at least three main sources of differentiated cells. Therefore, prevention of mesenchymal cell contamination was the first step in establishing a cultivation method for lacrimal gland epithelial cells. When CT was applied to the lacrimal gland culture medium, epithelial cells were successfully selected. Furthermore, adult (8-week-old) lacrimal gland epithelial cells could be cultured for 2–4 passages. We also used the sphere culture method as previously described [2]. Spheres were generated from single cells, not aggregates of cells, which were confirmed from mixed cultures of both GFP tagged and wild type cells. The spheres could be subcultured into secondary spheres, and cultivated on plastic dishes. The results support the hypothesis that lacrimal gland epithelial cells have unique progenitor cells. Therefore, lacrimal gland progenitor cells were further analyzed.

The cell lineage of a tissue at a specific developmental stage provides important information for characterizing tissue-specific stem cells. At the lacrimal gland developmental stage, budding of the gland originates from conjunctival epithelium, which branches and differentiates into mature tissue. This suggests that the ocular surface epithelial cell and the lacrimal gland are of the same origin, and their stem cells may have similar specific markers.

Ueda et al. [17] reported that mouse lacrimal gland primary cultures can proliferate in medium without serum. However, the multipotent potential to differentiate into three phenotypes, and then to proliferate and regenerate needs to be characterized to confirm the existence of stem cells in the lacrimal gland.

It was previously demonstrated that spheres generated from newborn mice, when transplanted into irradiated salivary glands, resulted in increased secretory function [1]. Furthermore, single mammalian cells selected by cell surface markers could generate cells with both luminal and myoepithelial lineages, and these generated functional lobuloalveolar units during pregnancy [2]. These data provide support for the existence of lacrimal gland stem cells. Although this study did not prove the existence of stem cells in the lacrimal gland, cells that form spheres from single cells are characteristic of stem cells, and all of the cells are epithelial-like. Further studies are necessary to confirm the existence of tissue-specific stem cells and their regenerative potential in the lacrimal gland.

In conclusion, newborn and adult lacrimal gland epithelial cells can be isolated successfully and subcultured in serum-free medium with CT. These cells undergo sphere formation from single cells and can be expanded and cultured on plastic dishes. A source of uniform lacrimal cultures should provide a useful model with which to study ocular disorders such as dry eye.
